# Persistent Hemarthrosis of the Knee after Arthroscopic Meniscal Repair

**DOI:** 10.1155/2023/8806299

**Published:** 2023-06-17

**Authors:** Shunichiro Kambara, Shinichi Yoshiya, Shintaro Onishi, Ryoji Yasumizu, Toshiya Tachibana

**Affiliations:** ^1^Department of Orthopaedic Surgery, JCHO Kobe Central Hospital, Hyogo, Japan; ^2^Department of Orthopaedic Surgery, Nishinomiya Kaisei Hospital, Hyogo, Japan; ^3^Department of Pathology, JCHO Kobe Central Hospital, Hyogo, Japan; ^4^Department of Orthopaedic Surgery, Hyogo College of Medicine, Hyogo, Japan

## Abstract

**Introduction:**

In this case report, we report a patient with complicated with persistent hemarthrosis following arthroscopic meniscal repair. *Case Presentation*. A 41-year-old male patient presented with persistent swelling of the knee 6 months after arthroscopic meniscal repair and partial meniscectomy performed for lateral discoid meniscal tear. The initial surgery was performed at another hospital. Four months after the surgery, swelling of the knee was noted when he resumed running. At his initial visit to our hospital, intra-articular blood accumulation was revealed via joint aspiration. A second arthroscopic examination performed 7 months after the initial procedure showed healing of the meniscal repair site and synovial proliferation. The suture materials identified during the arthroscopy were removed. Histological examination of the resected synovial tissue showed inflammatory cell infiltration and neovascularization. In addition, a multinucleated giant cell was identified in the superficial layer. After the second arthroscopic surgery, the hemarthrosis did not recur, and the patient was able to resume running without symptom one and a half years post-surgery.

**Conclusion:**

Bleeding from the proliferated synovia at or near the periphery of the lateral meniscus was thought to be the cause of the hemarthrosis as a rare complication following arthroscopic meniscal repair.

## 1. Introduction

As the surgical intervention for meniscal injuries, the meniscal repair has become a preferred procedure to prevent secondary damage to the cartilage after meniscal resection [1]. Although complications following the meniscal repair have been noted [2], satisfactory results with high healing rate have been documented in the literature [3]. As for adverse events related to fixation devices and materials, several complications such as inflammatory reaction to biodegradable polymer implants, cyst formation around the extra-articular anchor, and ganglion cyst generated by suture material have been reported [4–9]. However, as far as we know, persistent hemarthrosis has not been reported as a complication after meniscal repair surgery.

In this report, we present a case of persistent hemarthrosis after arthroscopic meniscal repair, which was successfully treated by removing the arthroscopic suture and tape material with synovectomy.

## 2. Case Presentation

A 41-year-old man (gymnastic class instructor) who underwent arthroscopic meniscal repair at another hospital 6 months before presented with pain and swelling in the left knee. At the initial surgery, a horizontal tear in the posterior and middle regions of the lateral discoid meniscus was identified, and combined partial meniscectomy (saucerization) and meniscal repair were performed. The surgical record documented moderate synovial hyperplasia without articular cartilage degeneration as additional intra-articular findings. In the meniscal repair procedure, the all-inside technique with Firstpass Mini® (Smith & Nephew plc, Watford, UK) device was employed for the repair in the posterior region applying two stitches of high-strength polyethylene tape (Ultratape, Smith & Nephew plc, Watford, UK), while the outside-in repair technique was utilized for the tear in the middle region applying two stitches of high-strength polyethylene suture (Ultrabraid, Smith & Nephew plc, Watford, UK). Although early postoperative recovery was uneventful, knee swelling was noted when the patient resumed running 4 months after surgery. Thereafter, the swelling persisted even after reducing the activity level, and joint aspiration was repeated several times in another clinic. Due to the persistent knee pain and swelling, the patient was unable to return to sports activities.

**Figure 1:**
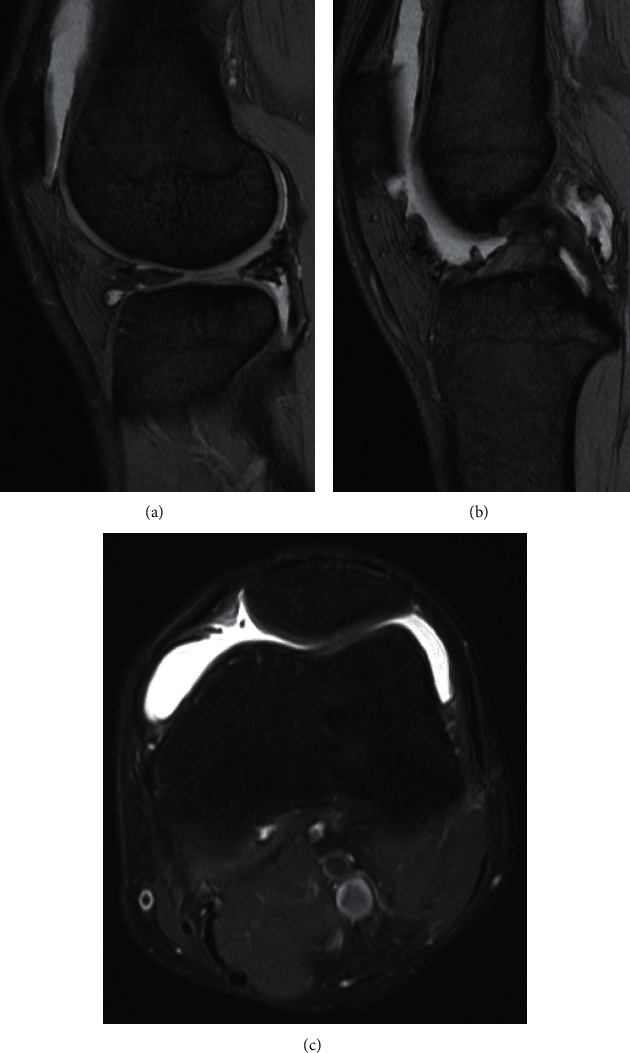
Magnetic Resonance Imaging (MRI) findings at 6 months after the initial surgery (T2-weighed sagittal images). (a) Repaired meniscus appears to be healed. (b) Synovial proliferation is identified around the posterior cruciate ligament and in the posterior compartment. (c) Intra-articular fluid retention is present.

Upon physical examination at the time of his first visit to our hospital (6 months after the initial surgery), the patient had pain, swelling, and localized warmth in the left knee. Flexion was restricted by 20–30° due to pain in the posterolateral aspect of the knee. Laboratory examination including blood coagulation tests showed no abnormalities, except for a slightly elevated C-reactive protein (CRP) (1.1 mg/dL reference range: 0.0–0.3 mg/dL). No inflammatory symptoms other than the left knee were noted. Joint aspiration revealed hemarthrosis with 20 ml of blood. At the next visit after 2 weeks, joint aspiration showed recurrent hemarthrosis with 40 ml of blood. Culture of the aspirated fluid was negative for bacteria. Radiological examination showed no osteoarthritic changes. On Magnetic Resonance Imaging (MRI), there were no intra- and extra-articular lesions such as osteochondral injury, hemangioma, or pigmented villonodular synovitis (PVNS) as a source of bleeding. The repaired meniscus appeared to be healed, while synovial proliferation was noted in the posterolateral compartment and around the posterior cruciate ligament (Figure 1).

**Figure 2:**
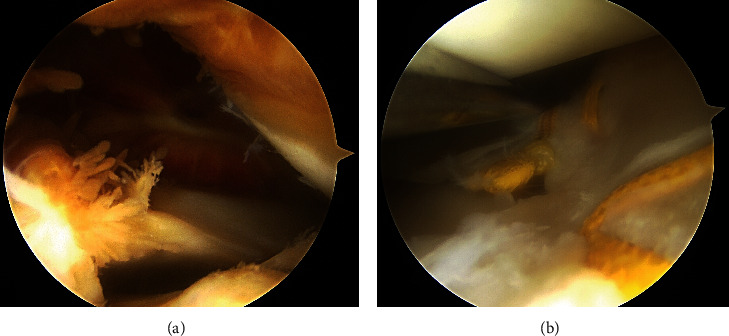
Arthroscopic findings. (a) Synovial proliferation with hemosiderin deposition is present in the posterolateral compartment. (b) Healing at the repair site is attained. The high-strength polyethylene tape and the knot are identified on the surface of the repaired meniscus in the posterolateral region.

Despite repeated joint aspiration and rest, hemarthrosis recurred and persisted. Considering the prolonged hemarthrosis, arthroscopy was conducted 7 months after the initial meniscal surgery. The surgery was performed with the use of a tourniquet to control bleeding during surgery. The arthroscopic examination revealed proliferation of brown-colored synovium, especially in the posterolateral compartment, but the repair site appeared to be completely healed (Figure 2). The suture material and the knots on the meniscal surface appeared to be intact without damage or failure (Figure 2(b)). In the subsequent arthroscopic procedure, synovectomy and coagulation with a radiofrequency device were performed for the area exhibiting the synovial proliferation. In addition, the tape and suture that remained visible on the meniscal surface were removed to eliminate any potential source of irritation. During and after the procedure, the intra-articular space was thoroughly irrigated with 12 liters of irrigation fluid.

**Figure 3:**
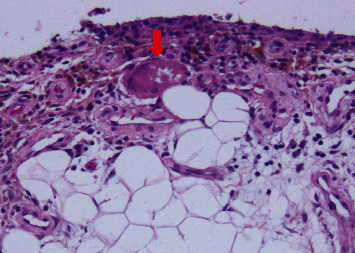
Photomicrograph of the resected synovium (HE staining). Infiltration of inflammatory cells with neovascularization, hemosiderin deposition, and a multinucleated giant cell containing transparent material (arrow) is detected.

Histopathological examination of the resected synovium revealed a moderate infiltration of inflammatory cells (predominantly lymphocytes) with neovascularization and hemosiderin deposition. A multinucleated giant cell containing a transparent substance was observed in the subepithelial stroma (Figure 3). Neither nodular accumulation of histiocyte-like atypical cells nor osteoclast type giant cells were observed.

Following the procedure, the knee was placed in a brace for a week with weight bearing permitted on the following day. The subsequent postoperative course was uneventful, and hemarthrosis did not recur. The patient resumed running 3 months after surgery and was able to return to his usual training activities such as running at full speed after 5 months. Two years after surgery, he remained asymptomatic, having achieved full range of motion without pain and maintaining a high level of activity.

## 3. Discussion

In this case, arthroscopic meniscal repair and a partial meniscectomy were performed for a lateral discoid meniscal tear, and hemarthrosis occurred without trauma 5 months later. Non-traumatic joint hemarthrosis may be caused by dysfunction of the hemostatic and coagulation systems (e.g., hemophilia), neoplastic lesions of the joint (e.g., PVNS, hemangioma, etc.), and spontaneous recurrent hemarthrosis. However, no coagulopathy or intra-articular hypervascular lesions were detected in this case.

Non-traumatic spontaneous recurrent hemarthrosis of the knee is documented in the literature [10–12]. Most cases are elderly patients with degenerative chondral and meniscal lesions in the lateral compartment. Furthermore, the significance of degenerative lateral meniscal tears as a cause of bleeding, including degenerative tears in the posterior horn of the lateral meniscus [10], arterial bleeding from the lateral meniscal rim due to degenerative tears [11], and bleeding from the peripheral arteries adjacent to a severely torn lateral meniscus [12], was reported. In this case, no hemorrhage was noted at the free edge of the lateral meniscus undergoing saucerization, while bleeding appeared to occur in the posterolateral compartment close to the meniscal rim neighboring the repair site. It was reported that the lateral inferior and middle genicular arteries supplying the lateral meniscus are much larger than the medial genicular artery and are located closer to the meniscal rim [13]. Therefore, injury or mechanical irritation to a peripheral artery at the repair site might have caused recurrent hemorrhage in the present case. Unlike previously reported cases of spontaneous knee hemarthrosis, there were no apparent osteoarthritic changes or meniscal degeneration. This case, therefore, presents a novel clinical feature from those documented in prior reports.

In addition to bleeding from degenerative lateral meniscal tears, mechanical insult or damage to the hypertrophied synovium in the osteoarthritic knee has been reported as another potential cause of spontaneous recurrent hemarthrosis [14, 15]. Burman et al. reported cases with hemarthrosis caused by bleeding from the synovium, which was subsequently resolved by synovectomy [15]. In our case, histological examination of the resected synovium revealed an infiltration of inflammatory cells with neovascularization and a multinucleated giant cell containing transparent material. These histological findings suggest that hypervascular synovial tissue as the result of a foreign body (debris from a polyethylene suture/tape) reaction may have been the cause of intra-articular bleeding. Postoperative pathological tissue reactions to fixation materials used in meniscal repair have been reported. These include foreign body reactions after all-inside meniscus repair using a biodegradable fixation device [7], cyst formation around the extra-articular anchor used in all-inside meniscal repair [5], and ganglion cysts around the polyester sutures after inside-out repair [8, 9]. Since polyethylene debris has been reported to induce adverse tissue reactions after total hip arthroplasty [16], the use of polyethylene tape or sutures in our case might have induced synovitis as the result of a foreign body reaction.

As discussed above, there are several possible causes accounting for recurrent and persistent hemarthrosis in the present case including bleeding from the degenerative lateral meniscus, the neighboring artery, or proliferated synovium. Since preoperative angiography was not performed and the arthroscopy was performed with tourniquet application, the exact site of bleeding was not pinpointed. Consequently, the findings observed in this case are not sufficient enough to determine the origin of the bleeding; however, it should be noted that recurrent hemarthrosis due to a foreign body reaction to suture could be encountered as a potential complication after arthroscopic meniscal repair.

Another notable finding, in this case, is that the posterolateral knee pain on deep flexion had been resolved after the tapes and sutures at the repair site were removed. It is known that the lateral meniscus moves significantly on flexion [17]. In addition, it has been reported that the inside-out repair suture bears a risk of entrapment of peri- and extraarticular soft tissues such as posterior capsule, lateral collateral ligament, and popliteus tendon [18, 19]. Too tight meniscal fixation in the present case may have over-constrained the lateral structures of the knee with secondary stretching and/or pinching on knee flexion.

## 4. Conclusion

In this report, we present a case of persistent hemarthrosis as a complication after combined arthroscopic meniscectomy and meniscal repair. Histological examination of the synovial sample revealed inflammatory cell infiltration as well as neovascularization and a multinucleated giant cell. Removal of the suture materials concomitant with the synovectomy and coagulation with a radiofrequency device successfully resolved the recurrent intra-articular hemorrhage.
